# Academic Buoyancy: Overcoming Test Anxiety and Setbacks

**DOI:** 10.3390/jintelligence11030042

**Published:** 2023-02-21

**Authors:** David William Putwain, Joost Jansen in de Wal, Thijmen van Alphen

**Affiliations:** 1 School of Education, Faculty of Arts Professional and Social Studies, Liverpool John Moores University, Liverpool L1 9DE, UK; 2 Research Institute of Child Development and Education, Faculty of Social and Behavioural Sciences, University of Amsterdam, Nieuwe Achtergracht, 127, 1018 WS Amsterdam, The Netherlands

**Keywords:** test anxiety, academic buoyancy, self-regulatory executive function model

## Abstract

High levels of test anxiety can be damaging for academic achievement, wellbeing, and mental health. It is important, therefore, to consider those psychological attributes that may offer protection against the development of test anxiety and its negative consequences, thereby contributing to a potential positive future life trajectory. Academic buoyancy, the ability to respond effectively to academic pressures and setbacks, is one such attribute that offers protection from high test anxiety. We begin by defining test anxiety and a brief review of the literature to consider the harmful nature of test anxiety. This is followed by a definition of academic buoyancy and brief review of the literature to consider the beneficial character of academic buoyancy. Next, we describe the Self-Regulatory Executive Function model of test anxiety and consider the mechanisms and processes by which academic buoyancy exerts beneficial effects on test anxiety. The paper concludes with a consideration of critical issues for the conceptualisation and measurement of academic buoyancy, arising from the synergies, connections, and relations, theorised with test anxiety, and how these may inform future studies.

## 1. Defining Test Anxiety

Test anxiety arises when a situation, in which a person’s performance will be evaluated, such as a test, is appraised as threatening (Spielberger and Vagg 1995). An emotional and affective reaction (often referred to as ‘tension’) is elicited comprising feelings of dizziness, a racing heart, shaking or trembling, stomach discomfort, and so on. These are accompanied by persistent worries about failure and its consequences, and a preoccupation with plans and solutions to all possible worst-case outcomes (often referred to as ‘worry’). Difficulties in concentration, problems in recall, and a sense of ‘going blank’ during a test (often referred to as ‘cognitive interference’), are also commonly experienced.

The aforementioned threat was originally described by Spielberger (1966) as ‘ego-threat’, the anticipation that one’s performance will be damaging for one’s self-view, self-image, or self-esteem (also see Baumeister et al. 2009). For instance, that failure would ruin educational plans or career ambitions, a view of oneself as being academic achiever, or result in negative judgements from others such as teachers, peers, or family members (Banks and Smyth 2015; Putwain 2009a). In these examples, failure is judged subjectively; for some students failure could be anything other than an ‘official’ pass grade. For other students it could be an aspired target grade (either from teachers or self-generated) or, for highly perfectionist students, anything other than the highest possible grade (Putwain 2009b).

Many forms of anxiety are characterised by a strong impetus to avoid the anxiety-eliciting object or event (Clark and Beck 2010). Test anxiety is no exception, although this motivation can manifest in markedly different ways; one can focus on avoiding the anxiety or the failure. One can avoid failure by expending effort in test preparation, being highly engaged in lessons, and using effective study- and test-taking strategies (Eysenck et al. 2007; Pekrun et al. 2004). This focus on avoiding failure can be adaptive for achievement in that it reduces the likelihood of failure or reduces the negative impact of anxiety on performance (e.g., Hardy et al. 2007; Putwain and Symes 2018). Many students, however, focus on avoiding anxious feelings by cognitively distancing themselves from the threat, distracting themselves, and engaging in alternative activities to examination preparation (Skinner and Saxton 2019; Stöeber 2004); using an English idiom, to ‘bury one’s head in the sand’. Alternatively, students could protect their self-esteem by ‘strategically’ withdrawing effort, or procrastinating starting test preparation, in order to deflect reasons of failure away from ability (Covington 2009; Martin et al. 2001, 2003). The focus on avoiding anxiety may provide short-term relief from unpleasant feelings of distress (e.g., Jensen et al. 2016). Over time, however, avoidance will increase the likelihood of failure due to missed opportunities for learning, examination preparation, and self-sabotage. Avoidance-based study behaviours commonly associated with test anxiety, such as procrastination and academic self-handicapping, negatively impact achievement (e.g., Kim and Seo 2015; Schwinger et al. 2014).

When defining test anxiety, it is important to make a clear distinction from exam stress as these terms may be used interchangeably in everyday parlance (Putwain 2007). That is, persons can use the term ‘stress’ to refer to feelings of distress which may include anxiety as well as other negative emotions, or the experience of being ‘under pressure’ which may include positive emotions (e.g., Folkman 2008).

In transactional models, events and situations are appraised as being irrelevant, benign, or stressful, in a primary appraisal (Blascovich 2008; Lazarus and Folkman 1984). In such models, irrelevant situations are those that have no relevance for the person. Benign situations are those with a positive outcome. Stressful situations are those that offer the potential for harm and loss on one hand, or for growth, mastery, and gain, on the other (Jamieson 2017; Jamieson and Hangen 2021). The person then appraises the coping resources and options available for responding to the stressful situation in a secondary appraisal. Challenge arises when persons believe they possess adequate resources to successfully respond to the stressful situation; threat arises when she or he believes they do not possess adequate resources (e.g., Skinner and Brewer 2002; Travis et al. 2020). Emotions are elicited from primary and secondary appraisals. Anxiety is one of several emotions, including hopelessness, and disappointment, that follow a threat appraisal. Stress, therefore, can refer to both challenge and threat whereas anxiety refers to a specific outcome arising from a threat appraisal (e.g., Folkman et al. 1985; Penley and Tomaka 2002).

### The Harmful Effects of Test Anxiety

Numerous studies have shown that test anxiety is negatively associated with academic achievement. In a landmark meta-analysis of 562 studies (1950 to 1986), Hembree (1988), found *r* = −0.24 for relations between aptitude/achievement tests scores and test anxiety from Grades 3 upwards. In a contemporary meta-analysis of 238 studies (1988 to 2016); von der Embse et al. (2018) found *r*s = −0.29 and −0.18, for relations between achievement test scores and the cognitive and emotional components of test anxiety, respectively. Furthermore, studies using longitudinal designs have shown that test anxiety negatively predicts subsequent achievement over and above the variance accounted for by prior achievement (Pekrun 1991, 1992; Putwain et al. 2015) and cognitive ability (Putwain et al. 2013).

Highly test anxious persons also report higher scores for indicators of emotion disorders (i.e., anxiety and depression). Studies are summarised in Table 1. Despite the inconsistency in approaches used to categorise groups of high- and low-test anxiety, four studies (Beidel and Turner 1988; Beidel et al. 1994; Herzer et al. 2014; King et al. 1995), showed that persons scoring highly on continuous measures of test anxiety met diagnostic thresholds for an anxiety disorder. One study (von der Embse et al. 2021) showed students scoring high on a continuous measure of test anxiety were at an elevated risk of developing generalized anxiety disorder (GAD) and panic disorder (PD) using pre-existing cut points on measures on the Revised Child Anxiety and Depression Scale (Chorpita et al. 2005). In addition, three studies (King et al. 1995; Warren et al. 1996; Weems et al. 2010) showed that persons scoring high on continuous measures of test anxiety also reported higher symptoms of emotional disorders (i.e., anxiety and depression).

These studies provide strong evidence for a link between test anxiety and emotional disorders, but do not consider the issue of directionality. Putwain et al. (2021a) showed, after controlling for school-related wellbeing (satisfaction and positive affect), in a study of 1198 participants in upper secondary education, that test anxiety and risk of developing an emotional disorder were reciprocally related; test anxiety, however, was a stronger predictor for the risk of developing a subsequent emotional disorder than vice versa. In addition, Putwain et al. (2021b) showed in a network analysis, based on a sample of 918 secondary school students, that indicators of test anxiety, GAD, and PD, formed distinct, but related communities. Test anxiety was not merely a symptom of GAD or PD.

There is also evidence that academic pressures, which highly test anxious persons are susceptible to, are related to a greater risk of suicide. Over a sixteen-month period in England, 2014–2015, Rodway et al. (2016) found that examination pressures were specifically cited in Coroners’ (a court official with the legal authority to hold an inquest into the cause of death) reports as a cause of adolescent suicide in 15% of cases. In a survey of 1455 undergraduate and postgraduate students at four North American universities, of the 9% of respondents that had contemplated, and the 1% who had attempted, suicide, academic problems (53%) were cited as the strongest reason (Furr et al. 2001). Although some may be tempted to downplay the severity of test pressures as being an ordinary part of schooling (Denscombe 2000), the aforementioned studies show that the consequences of test anxiety can be comparable to, and should therefore be treated with the same seriousness as, anxiety disorders (also see Gerwing et al. 2015).

## 2. Academic Buoyancy: Overcoming Test Anxiety and Setbacks

The use of high-stakes tests within education is ubiquitous (Shackleton 2014; Suto and Oates 2021). Persons differ widely in their responses to the pressures posed by such tests; some thrive whereas for others the experience can be highly anxiety provoking. The anxiety associated with tests, commonly referred to as test anxiety, is not merely a by-product of students who anticipate failure. Rather, test anxiety can interfere with cognitive processes, resulting in lower achievement (Owens et al. 2008). In addition, highly test anxious persons experience lower wellbeing and higher symptoms of emotional disorder (Putwain et al. 2021a). It is, therefore, critical to understand what psychological attributes can offer protection against test anxiety. In this paper, we consider a highly promising psychological attribute, that of academic buoyancy, the ability to effectively deal with typical educational adversities such as test pressures. Using the Self-Regulatory Executive Function model (Zeidner and Matthews 2005), we explore how academic buoyancy can positively impact the mechanisms and processes that underpin test anxiety. We then consider future research directions that would add value to further understanding how academic buoyancy can respond effectively to exam pressures.

### 2.1. Defining Academic Buoyancy

Academic buoyancy is defined as students’ ability to successfully deal with academic setbacks and challenges that are typical of the ordinary course of school life (Martin and Marsh 2008). Many students routinely experience challenges, setbacks, and pressures, during their schooling. Dips in motivation, feeling the pressures of high-stakes testing, managing multiple deadlines, facing difficult schoolwork, receiving lower grades or exam marks than hoped for or expected, and so on, are typical experiences for many students; they are not confined to a minority of vulnerable cases. Students differ in their ability to be able to deal with, and respond effectively to, these typical educational adversities. Some may struggle to deal with academic pressures and challenges and continue to experience difficulties and problems, whereas others will overcome these adversities and flourish; they are *buoyant* in the face of educational adversity. Academic buoyancy is an asset-orientated attribute that captures these differences referring to the successful navigation of *typical* educational adversities (Martin and Marsh 2008, 2009).

An important point of contrast when defining academic buoyancy is for the smaller number of students who experience major, intense, and long-lasting adversities, such as poverty, gang violence, chronic underachievement, bullying, school refusal, parental alcohol or drug abuse, learning disabilities, poor physical and mental health, and so on (e.g., Bellis et al. 2018; Felner and Devries 2013; Forber-Pratt et al. 2014). Despite the profound impact of such experiences, students thankfully can, and do, recover from these intense, and sometimes sustained, major adversities. Whereas buoyancy describes those students who can successfully overcome *typical* educational adversities, students who maintain their motivation, attendance, and educational achievement, when faced with problematic *major* adversities are described as educationally resilient (e.g., Condly 2006; Downey 2008).

Conceptually, Martin and Marsh (2008, 2009) argue, therefore, that buoyancy can be differentiated from resilience by means of degree (overcoming isolated patches of peer performance and types of school pressures requires buoyancy, whereas overcoming chronic underachievement and incapacitating levels of anxiety requires resilience) and kind (dips in motivation and engagement and dealing with negative feedback on one’s work requires buoyancy, whereas truancy, disaffection, and alienation from school, require resilience). Accordingly, buoyancy has relevance to the majority of students; resilience has relevance to the minority. In addition, resilience is an attribute required or developed once adversity presents in order to offset or manage risks to wellbeing. In contrast, academic buoyancy is a more proactive approach to managing typical educational adversities before they escalate. Accordingly, buoyancy is proposed at the ‘frontline’ of one’s academic development and progress, and resilience as the robust ‘backline’.

Only one study, thus far, has provided empirical evidence of the conceptual distinction between buoyancy and resilience. In a study of Australian secondary school students aged 11 to 19 years, Martin (2013) showed that when included in the same analytic model, academic buoyancy, but not resilience, predicted advantageous responses to typical school adversities (reduced anxiety, uncertain control, and failure avoidance), but not substantial school adversities. In contrast, resilience, but not school adversity, predicted beneficial responses to more substantial school adversities (disengagement and academic self-handicapping), but not typical school adversities.

### 2.2. The Beneficial Effects of Academic Buoyancy

There are a growing number of studies to show that academic buoyancy is associated with beliefs, emotions, and behaviours, considered to be beneficial for learning and academic achievement. Buoyancy has been shown to correlate positively with engagement, competence, effort, self-efficacy, planning, persistence, and pleasant achievement emotions (enjoyment, hope, and pride), and negatively with academic anxiety, test anxiety, and uncertain control, and unpleasant achievement emotions (anxiety, hopelessness, boredom, and shame), in samples of primary, secondary, and undergraduate students (Datu and Yang 2018; Hirvonen et al. 2019; Malmberg et al. 2013; Martin 2013; Mendez and Bauman 2018; Putwain et al. 2012; Ahmed Shafi et al. 2018). In addition, studies using longitudinal designs have shown that academic buoyancy predicts lower subsequent academic anxiety, test anxiety, uncertain control, and harmful school-related stress^1^, after controlling for autoregressive relations in samples of secondary school students (Hirvonen et al. 2020; Martin and Marsh 2008; Martin et al. 2013; Putwain et al. 2015).

Given the aforementioned relations between buoyancy and academically beneficial belief, affect, and behaviour, it is not unreasonable to anticipate that academic buoyancy would also predict achievement. Relations between buoyancy and achievement are, however, equivocal. Some studies have shown academic buoyancy is a positive predictor of academic achievement in secondary school (Martin 2014; Putwain et al. 2016) and undergraduate students (Yun et al. 2018). Other studies, however, have found statistically non-significant relations between buoyancy and achievement in secondary school (Collie et al. 2015; Putwain and Aveyard 2018) and undergraduate students (Fong and Kim 2019). Furthermore, in studies of primary school students, studies have shown that buoyancy is indirectly linked to achievement through academic self-concept (Colmar et al. 2019), or can moderate the negative relations between learning-related anxiety and achievement (Putwain et al. 2022b).

The inconsistency in these findings may be partly an artefact of buoyancy itself. Some highly buoyant students may have already ‘bounced back’ from previous periods of underachievement. Other highly buoyant students may be undergoing a period of underachievement at the time when achievement data were collected and are yet to ‘bounce back’. Thus, high academic buoyancy may not always be related to high achievement if measured in relatively close proximity. Some students may require time for beneficial belief, affect, and, behaviour, that buoyancy is associated with, to take effect. Indeed, studies that examine how academic buoyancy can overcome previous academic adversity are scarce. Two notable examples are Martin and Marsh (2009) and Putwain et al. (2021a).

Martin and Marsh (2020) showed, in a sample of secondary school students, that academic buoyancy moderated the positive relation between academic adversities measured one year apart. Academic adversities were defined as hardships and challenges, and included experiences of failure, not handing in assignments, being suspended from school, experiencing difficult relationships with peers and teachers, and so on. When academic buoyancy and adversity were high at the first measurement point, students experienced lower adversity at the second measurement point, compared to those with high adversity, but low buoyancy. In a sample of upper secondary school students, Putwain et al. (2021a) showed that academic buoyancy moderated relations between prior adversity (poor attendance and behaviour) and subsequent exam grades. When attendance was low, and poor behaviour high, the exam grades of those with high buoyancy was protected, relative to those with low buoyancy.

## 3. Test Anxiety and Academic Buoyancy

**Figure 1:**
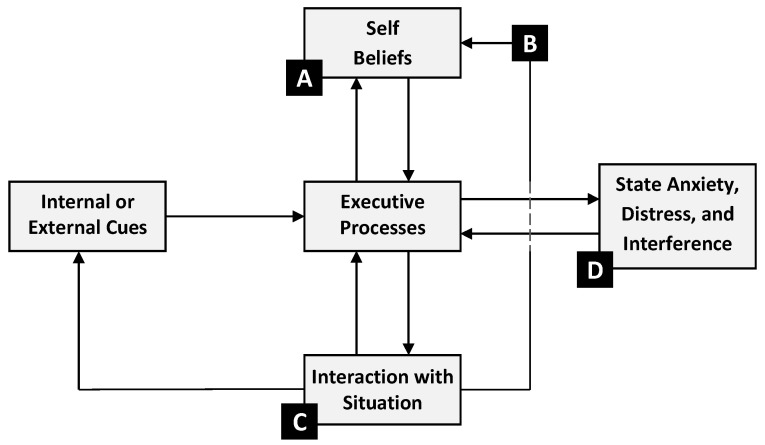
Points in the S-REF model where academic buoyancy could influence test anxiety processes.

The aforementioned studies have shown that academic buoyancy is negatively related to test (Putwain et al. 2012, 2016) and general academic anxiety (e.g., Martin 2013; Martin et al. 2010) in samples of secondary school students and that these relations are bidirectional (Martin and Marsh 2008; Martin et al. 2013; Putwain et al. 2015). That is, higher academic buoyancy predicts lower subsequent test anxiety and vice versa. Putwain and Daly (2013) proposed four points in the Self-Regulatory Executive Function model of test anxiety (S-REF: Zeidner and Matthews 2005; also see Putwain and Symes 2020; Putwain et al. 2022; see Figure 1) whereby academic buoyancy could impact processes to reduce test anxiety. Before we outline these points, however, we will briefly describe the S-REF model which comprises three inter-related systems: executive processes, self-beliefs, and person–situation interactions.

### 3.1. Systems Included in the S-REF Model

Executive processes refer to the conscious deliberative appraisal of the forthcoming test or exam. Appraisals include plans to deal with that test, the importance of the test, the likely consequences of failure, selecting the strategies and resources used to respond to, or cope with, the test, and metacognitions (i.e., how one’s internal state is monitored, and attempts to intensify or suppress certain beliefs). Executive processes are triggered by external or internal cues. An external cue could, for example, be a teacher reminding a student about a forthcoming examination; an internal cue could be students themselves recalling the date of a forthcoming examination. A test anxious response is more likely in persons who appraise a test to be high-stakes, where the perceived consequences of failure are high, and where the person uses coping strategies based on dealing with anxiety (emotion-focused and avoidance forms of coping) rather than the practical steps that can be taken to avoid failure. Steps, such as effort in exam preparation or ensuring one is using effective study- and test-taking skills, are referred to as problem-focused coping. Test anxious persons hold metacognitive beliefs (e.g., worry is dangerous) that can intensify anxiety and result in close monitoring of anxious thoughts and beliefs.

Self-beliefs refer to relative stable knowledge and perceptions of oneself, one’s future, and one’s relationships and environment, based on cognitive and affective appraisals. Executive processes draw on self-beliefs about one’s academic competence (e.g., academic self-concept, self-efficacy, and control), test-taking, and study-skills. Test anxiety is higher in persons with poor competence beliefs, who anticipate likely failure. Executive processes also draw on personal motivations. Highly test anxious persons are often motivated by a strong fear of failure; a need to not appear as incompetent, gain lower grades than peers, classmates, or family members, achieve lower than they have done previously, or lower than a target grade. Such motivations can be underpinned by a belief that failure indicates a lack of worth. The anticipation of failure, and the motivation to avoid failure, may result in a student reviewing their plans made for a test and ruminating on whether they are adequate or require changing. The focus on failure-related belief and emotion can feedback into executive processes to trigger emotion-focused and avoidance coping. The short-term feedback for executive processes, prompted by this reviewing and rumination, is attentionally demanding and can divert attention, and cognitive resources, away from other, potentially more useful, activities (e.g., test preparation).

Person–situation interactions refer to how executive processes influence, and in turn are influenced by, cognition and behaviour, in achievement settings (e.g., classroom, self-study, and examinations). As a result of being preoccupied with failure, test anxious persons become biased in their processing of threat-related information. This bias can show in vigilance to situational cues about poor competence and likely failure, or a misinterpretation of ambiguous cues in such a way to indicate likely failure or poor competence. These forms of attention bias magnify and maintain the perception of threat (Dong et al. 2017; Jastrowski et al. 2018; Putwain et al. 2011, 2020a; Zhang et al. 2018). Following on from emotion-focused or avoidance coping, the test anxious students can become academically disengaged and withdraw effort in lessons and test preparation. This may provide a reason for failure that protects self-worth against being judged as incompetent, but, paradoxically, increases the likelihood of failure due to missed learning and study opportunities. A common form of avoidance-based self-sabotage is procrastination. That is, delaying test preparation until it is too late. This interaction with the situation provides short-term feedback for executive processes, to maintain and reinforce the anticipation of failure, and long-term feedback for self-beliefs, to maintaining and reinforcing the belief that one is not competent.

The outcome of these processes is an increase in state anxiety, cognitive interference, and distress. Without any change, or intervention, the student continues to keep viewing tests as a threat, locked into a cycle of mutually supporting processes that maintain anxiety. The next time an internal or external cue triggers executive processes, the various S-REF processes are re-activated. Fortunately, the processes described in the S-REF model are changeable and interventions based on the S-REF model have been shown effective in reducing test anxiety (Putwain and Pescod 2018; Putwain and Symes 2020; Putwain and von der Embse 2021). As indicated by Putwain et al. (2015), academic buoyancy may capture a highly effective personal attribute that may protect a person from becoming test anxious, when subjected to academic pressures, thus enabling a person who has become test anxious to overcome that adversity, or offer protection for achievement against the damaging impact of test anxiety.

### 3.2. Academic Buoyancy and S-REF Model

The four points suggested by Putwain and Daly (2013) whereby academic buoyancy could positively impact on processes in the S-REF model to reduce test anxiety are shown in Figure 1. When an internal or external cue triggers executive processes signalling an evaluative situation, the highly buoyant student may access positive self-knowledge beliefs (point A). Positive self-knowledge beliefs could include appraisals of high competence beliefs (e.g., academic self-concept, self-efficacy, and control) and beliefs that one possesses good study and test-taking skills. In addition, the highly buoyant student accesses motivations based on task or self-judged standards (e.g., personal bests). In short, the belief of being able to handle the evaluative situation effectively (i.e., high buoyancy) reinforces. and is reinforced by high competence beliefs, outcome expectations, and a mastery orientation. Accordingly, the buoyant student has an expectation of success rather than failure and experiences lower test anxiety as a result of lowered threat perception (included within executive processes).

In the event that the highly buoyant student does receive failure feedback or lower than expected grades or marks, they can maintain their positive self-beliefs (point B). Failure feedback also acts as an internal cue for the appraisal process to reoccur in a continuing cycle of re-appraisal. The highly buoyant student may resolve to make plans for the future to ensure the likelihood for success, such as additional effort in examination preparation in the future and seeking help from teachers to establish where marks were lost. These plans trigger beneficial problem-focused forms of coping, rather than emotion-focused or avoidance coping, and maintain a success, gain-focused, orientation resulting in the buoyant student experiencing lower test anxiety.

In this example, buoyancy is a protective factor against the emergence of test anxiety through moderating (i.e., reducing) the impact of failure feedback on self-beliefs. Anxiety will not manifest because the student may think they are capable of handling the challenge (i.e., they expect success). The student will judge the evaluative situation as manageable and think of it as a chance to grow. Students may differ, however, in the rate at which they recover from their failure. This is likely to depend on the severity of failure, the psychoeducational environment (e.g., teacher–student relationship and a supportive academic environment at school), opportunities to develop and practice one’s skills, the timeliness of future assessments, and so on. All things being equal, we might expect buoyant responses to failure to be typified by a quicker recovery. Indeed, the speed of recovery may be an important definitional characteristic of buoyancy that has yet to be considered.

As a result of low use of emotion-focused or avoidance coping strategies, the highly buoyant student does not self-sabotage (i.e., disengage from studies, or withdraw effort in lessons and test preparation, as means of self-worth protection), or become biased towards threat (point C). Namely, the buoyant student’s response to failure is adaptive because of their belief of being in control. The maintained persistence in achievement-behaviours likely to result in success reinforces a lower perception of threat via a recursive feedback loop with executive processes. The use of strategy attributions for success and failure maintains self-beliefs of high control also via a recursive feedback loop. Thus, a virtuous cycle is created and the net result is that the highly buoyant student experiences less test anxiety.

Points A, B, and C, as described thus far, are truly proactive in that they prevent the highly buoyant student from becoming highly test anxious. However, there may still be circumstances whereby the highly buoyant student becomes test anxious. Reasons are multifarious, but could include excessive prolonged exposure to pressures from others, events in one’s life that continually trigger executive processes, and negative peer influence. The highly buoyant person could also possess metacognitive beliefs that intensify internal monitoring and suppress negative emotion in spite of the aforementioned virtuous cycle. The highly buoyant student would, however, be able to recover from their test anxiety more quickly and be less prone to the performance-interfering effects of test anxiety than a low buoyancy student (point D). For instance, the buoyant person who becomes anxious during an examination may be able to employ emotion regulation strategies that reduce anxiety quickly, draw on self-beliefs (e.g., “Even though I am anxious, I know that I have worked hard and I believe I could do well on this test”), or be benefitted by examination preparation to facilitate recall from memory even during periods of anxiety-induced interference with working memory capacity. We characterise this aspect of buoyancy as offering a quick ‘bounce back’ in the way that students can withstand the pressures of examinations.

Many of the aforementioned studies that evidence negative associations between academic buoyancy and test anxiety (Putwain et al. 2012, 2015; also see Putwain 2019), as well as those found for general academic anxiety (Hirvonen et al. 2020; Martin and Marsh 2008; Martin 2013; Martin et al. 2010, 2013), support the links theorized at points A, B, and C. With two notable exceptions, evidence for the proposed link at point D is more limited.

In a cluster analysis of 469 secondary school students, Putwain and Daly (2013) identified five profiles of students. Three of the profiles represented varying degrees of the inverse relation between test anxiety and academic buoyancy suggesting that academic buoyancy was related to lower test anxiety (i.e., consistent with the processes suggested for points A, B, and C). Two clusters, however, contained profiles of (1) mid-high test anxiety/mid buoyancy and (2) mid anxiety/high buoyancy. This is consistent with our theorizing that some academically buoyant students may still become high test anxious. In addition, examination performance of these clusters was higher than in clusters characterised by higher test anxiety in the absence of buoyancy. This implies that highly buoyant students were less prone to the performance interfering effects of test anxiety, thereby offering a ‘bounce back’ over the relatively short duration of an examination.

Putwain et al. (2015), in a sample of 325 secondary school students found a negative indirect relation between test anxiety and examination grade, mediated by a reduced use of problem-focused coping; the negative indirect relation, furthermore, was lessened at higher levels of buoyancy. The findings of Putwain and Daly (2013) and Putwain et al. (2015) provide initial evidence for the theorizing at point D that in the examination performance of highly buoyant students is protected when they become test anxious. A third study of *classroom*, rather than *test*, anxiety is also relevant here. In this study of 1242, primary school students, the protection offered by academic buoyancy for test performance declined with increasing anxiety (Putwain et al. 2022b). This may imply an age-related effect, whereby buoyancy may offer greater protection for examination performance in older students who have more strongly developed forms of emotion regulation strategies. It was also notable in this study, however, that academic buoyancy was measured one week before tests were taken; some highly buoyant students may not have had yet had the opportunity to recover. The quick ‘bounce back’ for test anxiety (point D in Figure 1) may be less relevant to classroom anxieties that are more strongly rooted in learning processes, requiring iterative, longer, cycles of feedback to benefit from the positive emotions, beliefs, and behaviours associated with academic buoyancy (i.e., engagement, control, planning, persistence, and self-efficacy; see Martin and Marsh 2008; Martin et al. 2010).

Although academic buoyancy is theorised to lower, or offer a quick ‘bounce back’ from, test anxiety partly through coping processes (i.e., less use of emotion-focused/avoidance and greater use of problem-focused, approaches), it is notable that studies have shown negligible correlations between academic buoyancy and coping (*r*s = −0.13 to 0.08; Putwain et al. 2012, 2015). This may be partly an artefact of the narrow range of coping approaches used in the aforementioned studies not capturing the strategies that students were using. Furthermore, it is possible that, as we suggest for achievement, some buoyant students are already implementing beneficial coping approaches, whereas others are yet to employ such strategies.

## 4. Future Research Directions

In the research on academic buoyancy that has been discussed in this paper, evidence is presented for the proactive protection that buoyancy offers against test anxiety among students (i.e., points A, B, and C in Figure 1). Less evidence exists for the suggested mitigating effects of academic buoyancy in students who—despite adaptive self-beliefs, executive processes, and person–situation interactions—still experience test anxiety (i.e., point D in Figure 1). As noted earlier, academically buoyant students are theorized to quickly ‘bounce back’ from their feelings of anxiety. However, to our knowledge, such a bounce back has never been explicitly investigated among students. Doing so in future research can explain why students with relatively high levels of anxiety and academic buoyancy still perform relatively well on exams (Putwain and Daly 2013; Putwain et al. 2015). That is, as suggested above, a quick bounce back that can protect students’ exam performance from negative effects of anxiety, because students high in this manifestation of buoyancy may not suffer from anxiety as long as students who are lower in buoyancy.

Studying academic buoyancy as students’ capacity to bounce back from peaks in anxiety would also further align the way academic buoyancy is conceptualized with how it is operationalized. That is, even though academic buoyancy is defined as students’ capacity to successfully deal with academic setbacks and challenges that are typical of the ordinary course of school life (Martin and Marsh 2008), it can be argued that existing research has not investigated students’ *capacity* to deal with typical setbacks and challenges related to school, but rather their *belief* in being able to do so. This is because, to date, academic buoyancy was almost exclusively studied through the academic buoyancy scale (ABS; Martin and Marsh 2008). This scale concerns a retrospective self-report questionnaire that asks students about their general (i.e., trait) ability to deal with typical challenges of ordinary academic life. Robinson and Clore (2002) explain that individuals, when filling out retrospective self-reports on traits, are likely to draw on their identity related beliefs (e.g., about how they deal with setbacks), rather than memories of episodes they actually went through. Although Martin and Marsh (2008, 2009) acknowledge that there is a need for objective and multidimensional indicants of buoyancy in addition to the ABS, this need has not yet been answered in research. Perhaps, it is therefore not surprising that the existing research primarily provides evidence for the impact of buoyancy on points A, B, and C in the S-REF model, as discussed in this paper. It is at these points that buoyancy as a belief would offer proactive protection against anxiety, whereas the effect of buoyancy as a reactive capacity to bounce back would be more salient at point D, after students have experienced anxiety to some extent.

Students’ reactive capacity to bounce back from typical challenges and setbacks that they encounter can be studied through inspecting fluctuations in anxiety after students have gone through anxiety evoking situations. These situations could be naturally occurring events in students’ everyday life at school, such as the announcement of an exam or receiving a poor test result. Studying how quickly students bounce back from such events would require time intensive (i.e., moment-to-moment or day-to-day) measures of their anxiety levels before and after the anxiety evoking situation.

An important first question to answer would be whether bouncing back from school related setbacks is typically a matter of minutes, hours, days or longer. To investigate such a question, questionnaires can now be distributed through the use of phone applications. Moreover, smartwatches can be used to monitor changes in heartrate and skin conductivity to represent physiological indicators of anxiety. Together, these methods allow us to more accurately assess the rate of recovery from anxiety.

If this rate has been determined, students who, within the established time-frame, return to their normal levels of anxiety relatively quickly can be said to be higher in their capacity to bounce back. Through analytical methods, aimed at modelling quick fluctuations over time, (i.e., dynamic structural equation modelling), the rate at which individuals return to a base-value can be captured by an auto-regressive parameter ‘φ’, also known to represent the inertia effect (McNeish and Hamaker 2019; Rottweiler and Nett 2021). This parameter varies between individuals and can be used as a predictor or outcome variable. As such, φ can be used to relate differences between students in their reactive manifestation of buoyancy to variables such as subsequent test performance. In addition to test performance, it would be interesting to relate differences in students’ capacity to bounce back from anxiety (φ) to other measures, because it could explain previous results, inform research about the nature of academic buoyancy, and provide insight in how the capacity to bounce back from anxiety can be stimulated. In particular, interesting measures would include (1) traditional (i.e., retrospective self-report) measures of anxiety and buoyancy, (2) time-intensive measures of buoyancy beliefs and the use of coping strategies, and (3) exposure to biofeedback interventions. Each is discussed below in more detail.

It would be interesting to relate differences in students’ capacity to bounce back from anxiety to traditional measures of anxiety and buoyancy because these differences can explain the demonstrated reciprocal negative relationships between trait buoyancy and anxiety (Martin et al. 2013; Putwain et al. 2015). This explanation, which has not been previously suggested in research, would comprise that in order to sustain high buoyancy beliefs over time, these beliefs have to be reinforced by successfully dealing with setbacks and challenges when these do result in anxiety (much like self-efficacy beliefs need mastery experiences in order to be sustained or enhanced; e.g., Joët et al. 2011). In other words, in the event that students experience anxiety, they also need to experience a quick bounce back (Point D in Figure 1) to allow them to continue to proactively employ their buoyancy beliefs to prevent adverse effects from setbacks and challenges in the future (points A, B, and C in Figure 1). If students do not experience such a quick bounce back from anxiety, their buoyancy beliefs may decrease and, as a consequence, they may experience anxiety more often. Reversely, higher buoyancy beliefs and fewer experiences of anxiety may result when students do experience a quick bounce back from setbacks.

Like bouncing back itself, these downward and upward spiral effects (Burns et al. 2008; Fredrickson and Joiner 2002) as a consequence of (not) recovering from anxiety quickly can be studied through time-intensive longitudinal designs (cf. Lavy and Eshet 2018). This would include collecting momentary data on buoyancy beliefs and anxiety for longer periods of time after students’ experience, and bounce back from anxiety (or not). Such measures can indicate to what extent a person’s buoyancy beliefs immediately suffer from not bouncing back quickly after anxiety is experienced. Moreover, time-intensive measures of buoyancy beliefs could show to what degree these beliefs vary within students from occasion to occasion in addition to being a stable individual difference between students. This could further inform research on the nature of academic buoyancy and whether it varies as a consequence of situational circumstances (such as anxiety evoking circumstances at school). Our own forthcoming research employed an adaptation of the work buoyancy scale (Martin and Marsh 2008) to measure teachers’ buoyancy beliefs from day to day. The results show factorial validity and reliability of this scale for measuring stable between-teacher differences as well as daily within-teacher variation in their buoyancy beliefs. The daily variations comprised 57.80% of the total variation in teachers’ buoyancy, which suggests that these beliefs vary substantially from day to day. Moreover, daily variations in teachers’ buoyancy beliefs were negatively related to daily variations in their anxiety. Because of reasons mentioned previously in this section, it would be interesting to replicate these findings among students.

Relating the rate at which students bounce back from peaks in anxiety to time intensive measures (e.g., ecological momentary assessments; Shiffman et al. 2008) of buoyancy beliefs and the use of coping strategies would allow researchers to further investigate the relationship between buoyancy and coping, and whether these are different concepts (cf., Putwain et al. 2012, 2015). It could be stated that bouncing back quickly after experiencing peaks in anxiety is part of being a buoyant student or follows from having high buoyancy beliefs. Alternatively, students who bounce back quickly can be considered to be good at coping with anxiety. Relating the rate at which students bounce back to measures of whether they were good at dealing with the setback that caused the anxiety (i.e., buoyancy beliefs) and what strategies they used to deal with it (coping) can provide clarity regarding the distinction between buoyancy and coping.

Finally, the rate at which students bounce back from anxiety may improve as a result of gaining metacognitive awareness of school or test anxiety and effects of trying to reduce it. Such awareness can be provided by real-time biofeedback, for example on heartrate variability (HRV). The HRV refers to the variation in time between successive heartbeats. HRV feedback interventions (e.g., increasing awareness of HRV or decreasing HRV through breathing exercises) have been shown to reduce anxiety, through increased self-regulation (Aritzeta et al. 2022; Goessl et al. 2017). It would be interesting to investigate how HRV biofeedback interventions relate to students’ capacity to bounce back from anxiety, as well as their proactive beliefs of being able to deal successfully with day-to-day setbacks in school.

In summary, we suggest that research and theorizing on academic buoyancy can be advanced through studying different manifestations of the concept by means of time-intensive longitudinal designs. The reactive capacity to bounce back from setbacks and challenges that academic buoyancy is theorized to include can be studied through momentary measures of anxiety. This reactive capacity potentially provides an explanation for existing research findings regarding the relationship between buoyancy and anxiety and their effects on exam performance, as well as the relationship between buoyancy and coping. Moreover, how quickly students bounce back from anxiety can explain their subsequent buoyancy beliefs and anxiety experiences. Studying these buoyancy beliefs on a momentary or daily basis can advance theorizing about the nature of buoyancy as well.

## 5. Conclusions

In this paper, we argue that academic buoyancy can offer protection against test anxiety through different mechanisms and processes based on the S-REF model of test anxiety (Zeidner and Matthews 2005). Three of these mechanisms (points A, B and C, in Figure 1) are proactive in that they prevent a person from becoming highly test anxious. The fourth (point D, in Figure 1) is conceptualised as offering a quick ‘bounce back’ for persons who have already become test anxious. The ability of students to ‘bounce back’ from test anxiety, or other academic adversities has yet to be studied. We round off the paper by considering how this aspect of academic buoyancy could be studied and, in doing so, the implications for further clarifying the nature of academic buoyancy.

## Figures and Tables

**Table 1:** Studies showing highly test anxious persons meet diagnostic thresholds for, and elevated indicators of, emotion disorder.

Study	Sample	Cut-Point	Findings
Beidel and Turner (1988)	3rd to 6th Grade	Test Anxiety Scale for Children (TASC) scores of ≥12 for boys and ≥16 for girls (HTA) and <7 or boys and <10 for girls (NTA)	HTA students met DSM-III criteria for social phobia (social anxiety disorder in DSM-5), overanxious disorder, specific phobia, or separation anxiety.
Beidel et al. (1994)	Mean ages of 10 (white sample) and 10.3 (African American sample) years	TASC scores of ≥12 for boys and ≥16 for girls (HTA) and <7 for boys and <10 for girls (NTA)	HTA students met DSM-III-R criteria for social phobia, overanxious disorder, or simple phobia.
King et al. (1995)	Grades 9 and 10	5th (NTA) and 95th (HTA) percentiles of TASC scores	HTA students reported higher scores on the Revised Children Manifest Anxiety Scale (RCMAS; *d*s = 0.90 to 1.67) than NTA students, and met DSM-III-R criteria for social phobia, simple phobia, and generalized anxiety disorder
Warren et al. (1996)	Grades 4, 6, and 10	33rd (NTA) and 66th (HTA) percentiles of Test Anxiety Inventory (TAI) scores	HTA students reported higher scores on the RCMAS (*d*s = 0.72 to 2.67) than NTA students.
Weems et al. (2010)	Grades 4 to 8	TASC scores of ≥8 (HTA) and <8 (NTA)	HTA students reported higher scores on the Revised Child Anxiety and Depression Scales (RCADS; *d*s = 0.71 to 0.95) than NTA students.
Herzer et al. (2014)	Mean ages of 25.3 (clinical sample) and 24.2 (control sample) years	German TAI score of ≥80	A German TAI score of ≥80 correctly identified 93.6% of the clinical sample (DSM-IV-TR diagnosis of specific phobia, social phobia, or depression).
von der Embse et al. (2021)	Years 10 to 13	Multidimensional Test Anxiety Scale (MTAS) scores of ≥58/60 (HTA)	Receiver operating characteristic curve analyses showed MTAS score of 58 met RCADS clinical threshold for generalised anxiety disorder and 60 met RCADS clinical threshold for panic disorder

Note. HTA = Highly test anxious, NTA = non-test anxious. Information about the scales and diagnostic criteria are included within Supplementary Materials File S1.

## Data Availability

No new data were created or analyzed in this study. Data sharing is not applicable to this article.

## Note

1 Items referred specifically to a threat appraisal (see Blascovich 2008; Folkman et al. 1985).

## Supplementary Materials

The following are available online at https://www.mdpi.com/article/10.3390/jintelligence11030042/s1, Supplementary Materials File S1: Details about the Scales Reported in Table 1. (American Psychiatric Association 1980, 1985, 1987, 2000; Hodapp 1996; Putwain et al. 2021c; Reynolds and Richmond 1978, 1979; Sarason et al. 1960) are cited in the Supplementary Materials.
