# Supplementary material for: Academic Buoyancy: Overcoming Test Anxiety and Setbacks
Source: J Intell. 2023 Feb 21;11(3):42. doi: 10.3390/jintelligence11030042 (PMC10056025; doi:10.3390/jintelligence11030042)
Supplement: Supplementary file 1 [file jintelligence-11-00042-s001.zip › jintelligence-2086691-supplementary.pdf]

- Supplementary Materials -

Details about the Scales Reported in Table 1:

1. The *Test Anxiety Scale for Children* (TASC: Sarason et al., 1960) is a 30-item unidimensional scale that require a binary *yes* or *no* response.
2. The *German Test Anxiety Inventory* (TAI-G: Hodapp, 1996) is a 30-item scale representing four components; worry (10 items), tension (8 items), interference (6 items), and lack of confidence (6 items). Participants respond on a four-point scale of *almost never* to *almost always*.
3. The *Multidimensional Test Anxiety Scale* (MTAS: Putwain et al., 2021) is a 16-item scale representing four components; worry (4 items), tension (4 items), cognitive interference (4 items), physiological indicators (4 items). Participants respond on a five-point scale of *strongly disagree* to *strongly agree*.
4. The *Revised Child Anxiety and Depression Scales* (RCADS: Chorpita et al., 2005) is a 47-item scale. Participants respond on a four-point scale of *never* to *always*. Subscales are provided for separation anxiety disorder (7 items), social phobia (9 items), generalized anxiety disorder (6 items), panic disorder (9 items), obsessive compulsive disorder (6 items), and major depressive disorder (10 items). Indicative cut-points for diagnostic risk are provided.
5. The *Revised Manifest Children's Anxiety Scale* (Reynolds et al., 1978) is 28-item scale accompanied by 9 lie items that require a binary *yes* or *no* response. A subsequent study (Reynolds & Richmond, 1979) indicated the scale comprised three factors. These were physiological manifestations of anxiety (9 items), worry and oversensitivity (10 items), and fear/concentration (9 items).

DSM-III Criteria for Separation Anxiety Disorder, Social Phobia, Specific Phobia, and Overanxious Disorder

Separation Anxiety Disorder (American Psychiatric Association, 1980, p. 52):

A. Excessive anxiety concerning separation from those to whom the child is attached, as manifested by at least three of the following:
   (1) unrealistic worry about possible harm befalling major attachment figures or fear that they will leave and not return
   (2) unrealistic worry that an untoward calamitous event will separate the child from a major attachment figure, e.g., the child will be lost, kidnapped, killed, or be the victim of an accident
   (3) persistent reluctance or refusal to go to school in order to stay with major attachment figures or at home
   (4) persistent reluctance or refusal to go to sleep without being next to a major attachment figure or to go to sleep away from home
   (5) persistent avoidance of being alone in the home and emotional upset if unable to follow the major attachment figure around the home
   (6) repeated nightmares involving theme of separation
   (7) complaints of physical symptoms on school days, e.g., stomachaches, headaches, nausea, vomiting
   (8) signs of excessive distress upon separation, or when anticipating separation, from major attachment figures, e.g., temper tantrums or crying, pleading with parents not to leave (for children below the age of six, the distress must be of panic proportions)
   (9) social withdrawal, apathy, sadness, or difficulty concentrating on work or play when not with a major attachment figure

B. Duration of disturbance of at least two weeks.

C. Not due to a Pervasive Developmental Disorder, Schizophrenia, or any other psychotic disorder.

D. If 18 or older, does not meet the criteria for Agoraphobia.

Social Phobia (American Psychiatric Association, 1980, p. 228):

A. A persistent, irrational fear of, and compelling desire to avoid, a situation in which the individual is exposed to possible scrutiny by others and fears that he or she may act in a way that will be humiliating or embarrassing.

B. Significant distress because of the disturbance and recognition by the individual that his or her fear is excessive or unreasonable.

C. Not due to another mental disorder, such as Major Depression or Avoidant Personality Disorder

Specific Phobia (American Psychiatric Association, 1980, p. 229-230):

A. A persistent, irrational fear of, and compelling desire to avoid, an object or a situation other than being alone, or in public places away from home (Agoraphobia), or of humiliation or embarrassment in certain social stations (Social Phobia). Phobic objects are often animals, and phobic situations frequently involve heights or closed spaces.

B. Significant distress from the disturbance and recognition by the individual that his or her fear is excessive or unreasonable.

C. Not due to another mental disorder, such as Schizophrenia or Obsessive Compulsive Disorder.

Overanxious Disorder (American Psychiatric Association, 1980, pp. 56-57):

A. The predominant disturbance is generalized and persistent anxiety or worry (not related to concerns about separation), as manifested by at least four of the following:

(1) unrealistic worry about future event
(2) preoccupation with the appropriateness of the individual's behavior in the past
(3) overconcern about competence in a variety of areas, e.g., academic, athletic, social
(4) excessive need for reassurance about a variety of worries
(5) somatic complaints, such as headaches or stomachaches, for which no physical basis can be established
(6) marked self-consciousness or susceptibility to embarrassment or humiliation
(7) marked feelings of tension or inability to relax

B. The symptoms in A have persisted for at least six months.
C. If 18 or older, does not meet the criteria for Generalized Anxiety Disorder.
D. The disturbance is not due to another mental disorder, such as Separation Anxiety Disorder, Avoidant Disorder of Childhood or Adolescence, Phobic Disorder, Obsessive Compulsive Disorder, Depressive Disorder, Schizophrenia, or a Pervasive Developmental Disorder.

DSM-IIIR Criteria for Social Phobia, Simple Phobia, and Overanxious Disorder

Social Phobia (American Psychiatric Association, 1987, pp. 242-243):

A. A persistent fear of one or more situations (the social phobic situations) in which the person is exposed to possible scrutiny by others and fears that he or she may do something or act in a way that will be humiliating or embarrassing. Examples include: being unable to continue talking while speaking in public, choking on food when eating in front of others, being unable to urinate in a public lavatory, hand-trembling when in the presence pf others, and saying foolish things or not being able to answer questions in social situations.
B. In another Axis II or Axis I disorder is present, the fear in A is unrelated to it, e.g., the fear is not of having a panic attack (Panic Disorder), stuttering (Stuttering), trembling (Parkinson's disease), or exhibiting abnormal eating behavior (Anorexia Nervosa or Bulimia Nervosa).
C. During some phase of the disturbance exposure to public scrutiny stimulus (or stimuli) almost invariable provokes an immediate anxiety response.
D. The phobic situation is avoided, or endured with intense anxiety.
E. The avoidant behaviour interferes with occupational functioning or with usual social activities or relationships with others, or there is marked distress about having the fear.
F. The person recognizes his or her fear as excessive and unreasonable.
G. If the person is under 18, the disturbance does not meet the criteria for Avoidant Disorder of Childhood or Adolescence.

Specify generalized type if the phobic situation includes most social situations, and also consider the additional diagnosis of Avoidant Personality Disorder

Simple Phobia (American Psychiatric Association, 1987, p. 244-245):

A. A persistent fear of a circumscribed stimulus (object or situation) other than fear of having a panic attacked (as in Panic Disorder) or of humiliation or embarrassment in social situations (as in Social Phobia).
   Note: Do not include fears that are part of Panic Disorder or Agoraphobia or Agoraphobia without History of Panic Disorder
B. During some phase of the disturbance, exposure to the specific phobia stimulus (or stimuli) almost invariable provokes an immediate anxiety response.
C. The object or situation is avoided, or endured with intense anxiety.
D. The fear or avoidant behaviour significantly interferes with the person's normal routine or usual social activities or relationships with others, or there is marked distress about having the fear.
E. The person recognizes his or her fear as excessive and unreasonable.
F. The phobic stimulus is unrelated to the content of the obsessions of Obsessive Compulsive Disorder or the trauma of Post-Traumatic Stress Disorder

Overanxious Disorder (American Psychiatric Association, 1987, pp. 64-65):

A. Excessive or unrealistic anxiety or worry for a period of six months or longer, as indicated by frequent occurrence of at least four of the following:
   (1) excessive or unrealistic worry about future events
   (2) excessive or unrealistic concern about the appropriateness of past behavior
   (3) excessive or unrealistic concern about competence in one or more areas, e.g., athletic, academic, social
   (4) somatic complaints, such as headaches or stomachaches, for which no physical basis can be established
   (5) marked self consciousness
   (6) excessive need for reassurance about a variety of concerns
   (7) marked feelings of tension or inability to relax

B. If another Axis I disorder is present (e.g., Separation Anxiety Disorder, Phobic Disorder, Obsessive Compulsive Disorder), the focus of the symptoms in A are not limited to it. For example, if Separation Anxiety Disorder is present, the symptoms in A are not exclusively about separation. In addition, the disturbance does not occur only during the course of a psychotic disorder or Mood Disorder.
C. If 18 or older, does not meet the criteria for Generalized Anxiety Disorder.
D. Occurrence not exclusively during the course of a Pervasive Developmental Disorder, Schizophrenia, or any other type of psychotic disorder.

DSM-IV-TR Criteria for Social Phobia, Specific Phobia, and Depression

Social Phobia (American Psychiatric Association, 2000, pp. 456):

A. A marked and persistent fear of one or more social or performance situations in which the person is exposed to unfamiliar people or to possible scrutiny by others. The individual fears that he or she will act in a way (or s how anxiety symptom s) that will be humiliating or embarrassing. Note: In children, there must be evidence of the capacity for age-appropriate social relationships with familiar people and the anxiety must occur in peer settings, not just in interactions with adults.
B. Exposure to the feared social situation almost invariably provokes anxiety, which may take the form of a situationally bound or situation ally predisposed Panic Attack. Note: In children, the anxiety may be expressed by crying, tantrum s, freezing, or shrinking from social situations with unfamiliar people.
C. The person recognizes that the fear is excessive or unreasonable. Note: In children, this feature may be absent.
D. The feared social or performance situations are avoided or else are endured with intense anxiety or distress.
E. The avoidance, anxious anticipation, or distress in the feared social or performance situation(s) interferes significantly with the person 's normal routine, occupational (academic) functioning, or social activities or relationships, or there is marked distress about having the phobia.
F. In individuals under age 18 years, the duration is at least 6 months.
G. The fear or avoidance is not due to the direct physiological effects of a substance (e.g., a drug of abuse. a medication) or a general medical condition and is not better accounted for by another mental disorder (e.g., Panic Disorder With or Without Agoraphobia. Separation Anxiety Disorder, Body Dysmorphic Disorder, a Pervasive Developmental Disorder, or Schizoid Personality Disorder).
H. If a general medical condition or another menta l disorder is present, the fear in Criterion A is unrelated to it, e.g., the fear is not of Stuttering, trembling in Parkinson's disease, or exhibiting abnormal eating behavior in Anorexia Nervosa or Bulimia Nervosa.

Specify if: Generalized: if the fears include most social situations (also consider the additional diagnosis of Avoidant Personality Disorder)

Specific Phobia (American Psychiatric Association, 2000, pp. 449-450):

A. Marked and persistent fear that is excessive or unreasonable, cued by the presence or anticipation of a specific object or situation (e.g., flying, heights, animals, receiving an injection, seeing blood).
B. Exposure to the phobic stimulus almost invariably provokes an immediate anxiety response, which may take the form of a situationally bound or situationally predisposed Panic Attack. Note: In children, the anxiety may be expressed by crying, tantrums, freezing, or clinging.
C. The person recognizes that the fear is excessive or unreasonable. Note: In children, this feature may be absent.
D. The phobic situation(s) is avoided or else is endured with intense anxiety or distress.
E. Th e avoidance, anxious anticipation, or distress in the feared situation(s) interferes significantly with the person's normal routine, occupational (or academic) functioning, or social activities or relationships, or there is marked distress about having the phobia.
F. In individuals under age 18 years, the duration is at least 6 months.
G. The anxiety, Panic Attacks, or phobic avoidance associated with the specific object or situation are not better accounted for by another mental disorder, such as Obsessive-Compulsive Disorder (e.g., fear of dirt in someone with an obsession about contamination) , Posttraumatic Stress

Disorder (e.g., avoidance of stimuli associated with a severe stressor), Separation Anxiety Disorder (e.g., avoidance of school), Social Phobia (e.g., avoidance of social situations because of fear of embarrassment), Panic Disorder With Agoraphobia, or Agoraphobia Without History of Panic Disorder.

Specify type:
Animal Type
Natural Environment Type (e.g., heights, storms, water)
Blood - Injection-Injury Type
Situational Type (e.g., airplanes, elevators, enclosed places)
Other Type (e.g., fear of choking, vomiting, or contracting an illness; in children, fear of loud sounds or costumed characters

Major Depressive Disorder (American Psychiatric Association, 2000, pp. 356):

A. Five (or more) of the following symptoms have been present during the same 2-week period and represent a change from previous functioning; at least one of the symptoms is either (l) depressed mood or (2) loss of interest or pleasure.

Note: Do not include symptoms that are clearly due to a general medical condition, or mood-incongruent delusions or hallucinations.

(1) depressed mood most of the day, nearly every day, as indicated by either subjective report (e.g., feels sad or empty) or observation made by others (e.g., appears tearful). Note: In children and adolescents, can be irritable mood.
(2) markedly diminished interest or pleasure in all, or almost all, activities most of the day, nearly every day (as indicated by either subjective account or observation made by others)
(3) significant weight loss when not dieting or weight gain (e.g., a change of more than 5% of body weight in a month), or decrease or increase in appetite nearly every day. Note: In children, consider failure to make expected weight gains.
(4) insomnia or hypersomnia nearly every day
(5) psychomotor agitation or retardation nearly every day (observable by others, not merely subjective feelings of restlessness or being slowed down)
(6) fatigue or loss of energy nearly every day
(7) feelings of worthlessness or excessive or inappropriate guilt (which may be delusional) nearly every day (not merely self-reproach or guilt about being sick)
(8) diminished ability to think or concentrate, or indecisiveness, nearly every day (either by subjective account or as observed by others)
(9) recurrent thoughts of death (not just fear of dying), recurrent suicidal ideation without a specific plan, or a suicide attempt or a specific plan for committing suicide
(10)

B. The symptoms do not meet criteria for a Mixed Episode.
C. The symptoms cause clinically significant distress or impairment in social, occupational. or other important areas of functioning.
D. The symptoms are not due to the direct physiological effects of a substance (e.g., a drug of abuse, a medication) or a general medical condition (e.g., hypothyroidism).
E. The symptoms are not better accounted for by Bereavement, i.e., after the loss of a loved one, the symptoms persist for longer than 2 months or are characterized by marked functional impairment, morbid preoccupation with worthlessness, suicidal ideation, psychotic symptoms or psychomotor retardation.

References

1. American Psychiatric Association. (1980). *Diagnostic and Statistical Manual of Mental Disorders (3rd Edition)*. American Psychiatric Association.
2. American Psychiatric Association. (1985). *Diagnostic and Statistical Manual of Mental Disorders (3rd Edition Revised)*. American Psychiatric Association.
3. American Psychiatric Association. (2000). *Diagnostic and Statistical Manual of Mental Disorders (4th Edition Text Revised)*. American Psychiatric Association.
4. Chorpita, B. F., Moffitt, C., & Gray, J. (2005). Psychometric properties of the Revised Child Anxiety and Depression Scale in a clinical sample. *Behaviour Research and Therapy, 43*, 309-322. https://doi.org/10.1016/j.brat.2004.02.004
5. Hodapp, V. (1996). The TAI-G: A multidimensional approach to the assessment of test anxiety. In C. Schwarzer & M. Zeidner (Eds.), *Stress, anxiety and coping in academic settings* (pp. 95-130). Francke Verlag.
6. Putwain, D.W., von der Embse, N.P., Rainbird, E.C., & West, G. (2021). The development and validation of a new Multidimensional Test Anxiety Scale (MTAS). *European Journal of Psychological Assessment, 37*(3), 236-246. https://doi.org/10.1027/1015-5759/a000604
7. Reynolds, C. R., & Richmond, B. (1978). What I think and feel: A revised measure of children's manifest anxiety. *Journal of Abnormal Child Psychology, 6*(2), 271–280. https://doi.org/10.1023/A:1025751206600
8. Reynolds, C. R., & Richmond, B. O. (1979). Factor structure and construct validity of
9. 'What I think and feel': The revised children's manifest anxiety scale. *Journal of Personality Assessment, 43*(3), 281–283. https://doi.org/10.1207/s15327752jpa4303_9
10. Sarason, S. B., Davidson, K. S., Lighthall, F. F., Waite, R. R., & Ruebush, B. K. (1960). *Anxiety in elementary school children.* Wiley.
